# Gastric cancer metastasis-related NT5DC2 indicates unfavorable prognosis of patients

**DOI:** 10.1097/MD.0000000000035030

**Published:** 2023-10-06

**Authors:** Rula Sha, Jiaming Zhang, Fanjie Meng, Getu Zhaori

**Affiliations:** a Department of Internal Medicine-Oncology, Inner Mongolia Autonomous Region People’s Hospital, Hohhot, Inner Mongolia, P.R. China; b Department of Internal Medicine, Inner Mongolia Medical University, Hohhot, Inner Mongolia, P.R. China; c Department of Abdominal Surgery, The Affiliated People’s Hospital of Inner Mongolia Medical University, Hohhot, Inner Mongolia, P.R. China.

**Keywords:** gastric cancer, metastasis, NT5DC2, prognosis

## Abstract

**Purpose:**

Approximately 80 to 90% of patients with gastric cancer (GC) eventually develop into metastatic GC nowadays,because GC is difficult to be diagnosed at an early stage. GC patients with metastases typically have a poor prognosis. It is necessary to explore a potential prognostic marker in metastatic GC.

**Methods:**

All GC data were obtained from The Cancer Genome Atlas and Gene Expression Omnibus databases. The metastasis-related candidate gene and its role in GC were analyzed by comprehensive analysis.

**Results:**

Totally 1049 metastasis-related genes were identified in GC. Univariate Cox regression analysis screened the top 10 genes (PDHX, SLC43A1, CSAG2, NT5DC2, CSAG1, FMN1, MED1, HIVEP2, FNDC3A, and PPP1R2) that were closely correlated with prognosis of GC patients. Among which, NT5DC2 was screened as the target gene for subsequent study. The NT5DC2 expression were increased in primary GC and metastatic GC samples. Moreover, GC patients with high NT5DC2 expression exhibited shorter overall survival and post progression survival, and the NT5DC2 was metastatic GC patients’ independent prognostic factor. Totally 29 pathways were activated in metastatic GC samples with high NT5DC2 expression. Four immune cells’ infiltration were significantly different between NT5DC2 high and low expressed metastatic GC patients. NT5DC2 showed significantly negative correlations with 6 types of immune cells’ critical marker genes and 5 types of immune cell infiltration. The 10 immune checkpoint expressions were decreased in high NTDC2 expression metastatic GC patients.

**Conclusions:**

NT5DC2 plays a prognostic role in metastatic GC. GC patients with high NT5DC2 expression indicates unfavorable prognosis.

## 1. Introduction

As known to all, gastric cancer (GC) has a high degree of malignancy, which is the fifth most commonly malignant cancer. And GC also is the third leading cause of cancer-related death throughout the world; moreover, the morbidity of GC is usually higher in Eastern Asia.^[1]^ GC patients’ survival rate remains low worldwide partially because resection is applicable only in early-stage patients. However, 80 to 90% of GC patients who are diagnosed at a late stage, resulting in a poor prognosis and metastases.^[2,3]^ Obviously, under the current treatment options and earlier detection strategies, GC remains a fatal disease that is uncontrolled.^[4]^ GC patients with the distant metastasis and the invasive infiltration usually have a poor prognosis, which is a significant challenge for biologists as well as clinicians.^[5,6]^ The traditional bulk-leveled methods do not focus on the role of subpopulations, so the mechanisms of GC metastasis are still elusive.^[7]^ In recent years, there has been considerable evidence that aberrant genes and pathways might be involved in metastatic GC development based on genomic analyses.^[8,9]^ An accumulating body of studies focus on the role of metastases in GC, whereas the underlying details between metastasis and prognosis of GC patients are still largely unclear.

As an oncogene in various tumors, NT5DC2 has a haloacid dehalogenase (HAD) motif in its N-terminus.^[10]^ It is worth pointing out that a number of phosphatases of the HAD superfamily may contribute to the development of cancer by promoting cell proliferation and migration et al. And in all organisms, the HAD family members are quantitatively dominated by phosphatases (79%).^[11]^ In previous studies, it has been shown that proteins in the NT5DC family bear a HAD motif, but their physiological role remains unclear.^[12]^ Recent research has proved that NT5DC2 can be a biomarker of prognosis or target of treatment for different cancers. In addition, NT5DC2 has been proved to be involved in the metastases of these cancers.^[13–15]^ Nevertheless, the role of NT5DC2 in GC prognosis and the correlation between NT5DC2 and metastasis in GC remain unknown.

This research aimed to explore target genes associated with metastasis in GC and investigated the potential impacts of target gene on the prognosis of GC patients using bioinformatics techniques. We identified NT5DC2 is a key GC metastatic related gene. The GC patients with high NT5DC2 expression indicates unfavorable prognosis. Our research will help to the identification of a new prognostic marker for metastatic GC.

## 2. Material and methods

### 2.1. Data resources

The mRNA expression profiles and corresponding clinical information of GC patients were extracted from The Cancer Genome Atlas (TCGA, https://tcga-data.nci.nih.gov/tcga/) database, among which 381 patients had complete survival information. According to the clinical information, 15 metastatic GC samples and 31 adjacent samples were selected.

Based on the Gene Expression Omnibus (GEO, https://www.ncbi.nlm.nih.gov/geo/), we obtained 5 datasets. These datasets were respectively GSE49051, GSE118916, GSE54129, GSE57303, and GSE14208. GSE49051 contained 3 GC samples and 3 normal samples. GSE118916 consisted of 15 GC samples and 15 normal samples. GSE54129 included 11 GC samples and 21 normal samples. GSE57303 and GSE14208 contained 70 GC samples and 123 metastatic GC samples, respectively.

Ethical approval was not required in this study because the data used in this work were collected from public database and all analyses were performed using bioinformatics tools.

### 2.2. Differential expression analysis

We used the limma package of R language (version 4.2.1, same as below) for the differential expression analysis. Only those differentially expressed genes (DEGs) with |Log2FC| >0.5 and FDR <0.05 were considered significant.

### 2.3. Survival analysis

Based on the Kaplan–Meier (KM) method, the survival analysis was conducted using the survival and survminer package of R language. Multivariate Cox regression analysis was undertaken to determine whether NT5DC2 was an independent prognostic indicator for GC.

### 2.4. Gene set enrichment analysis

Gene Set Enrichment Analysis (GSEA) was conducted based on the gene set c2.cp.kegg.v2022.1.Hs.symbols.gmt in the Molecular Signatures Database (MSigDB) (software version: 4.2.3, screening criteria: NOM *P* < .05).

### 2.5. Immune cell infiltration

The relative proportions of 22 tumor-infiltrating immune cells in each sample were calculated in CIBERSORT software.^[16]^ CIBERSORT was used to characterize the composition of infiltrating immune cells by the 547 preset barcode genes according to the deconvolution algorithm. This was done on the basis of the gene expression matrix. The sum of all relative infiltrating proportions of estimated immune cells in each sample was equal to 1.

### 2.6. Statistical analyses

Gene expression differences among different groups were determined using the Wilcoxon rank-sum test. The statistically significant criterion was *P* < .05. All statistical analyses were performed with R software version 4.2.1.

## 3. Results

### 3.1. Key gene NT5DC2 was related to GC metastases

**Figure 1:**
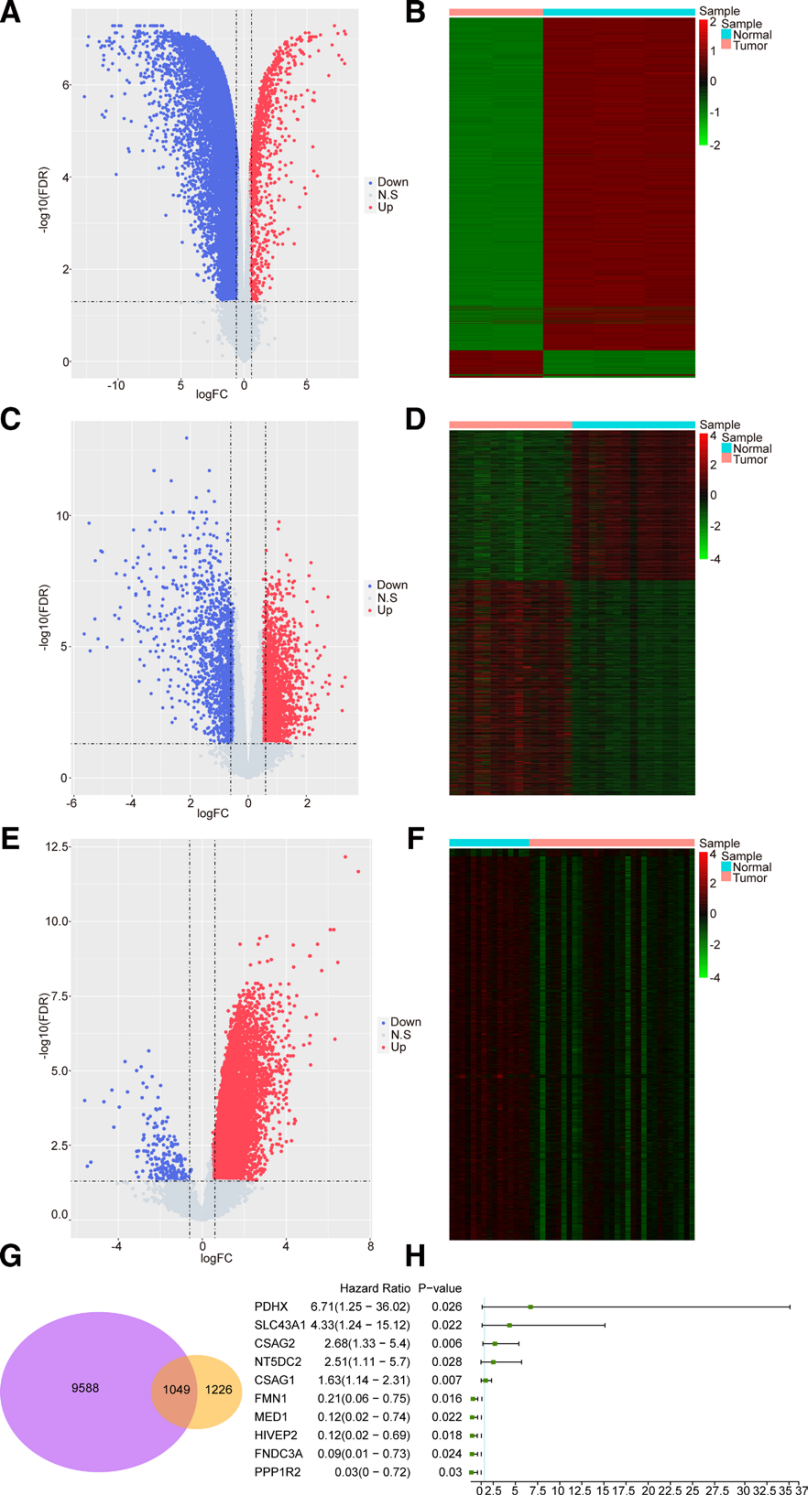
NT5DC2 was related to GC metastases. (A,B) DEGs identified between primary GC samples compared with the normal samples in GSE49051 and the result of heatmap. (C,D) DEGs identified between primary GC samples compared with the normal samples in GSE118916 and the result of heatmap. (E,F) DEGs identified between metastatic GC samples and the control samples in TCGA database and the result of heatmap. (G) The result of 1049 potential metastasis-associated candidate genes. (H) Top 10 genes which were closely correlated with the survival of GC patients are shown on a univariate Cox regression analysis forest plot. DEGs = differentially expressed genes, GC = gastric cancer.

First, we performed differential expression analyses in GSE49051 and GSE118916 datasets, respectively. In GSE49051 dataset, compared with normal samples, 1143 up-regulated DEGs and 13,999 down-regulated DEGs were obtained in primary GC samples. In total, 15,142 DEGs were identified (Fig. 1A,B). In GSE118916 dataset, we have identified 3667 DEGs in primary GC samples compared with the normal samples, of which 2159 DEGs were up-regulated and 1508 DEGs were down-regulated (Fig. 1C,D); 2275 DEGs were obtained from cross-analysis between 5142 DEGs and 3667 DEGs, which were related to GC development.

Second, we performed the same analyses in TCGA database. Compared with the adjacent samples, 10,425 up-regulated DEGs and 212 down-regulated DEGs were identified in metastatic GC samples, for a total of 10,637 DEGs (Fig. 1E,F), which were correlated with the metastases of GC. There were 1049 potential metastasis-associated candidate genes obtained from the intersection of 2275 DEGs and 10,637 DEGs (Fig. 1G). The details of results were listed in Table S1 (Supplemental Digital Content, http://links.lww.com/MD/J814, which illustrates all 1049 candidate genes).

Subsequently, based on these 1049 potential metastasis-associated candidate genes, an univariate Cox regression analysis was conducted to further identify the prognosis-related gene in metastatic GC. Finally, the top 10 most significant prognosis-related genes in GC patients were showed in Figure 1H. NT5DC2 was screened for our subsequent analyses.

### 3.2. Up-regulated NT5DC2 was observed in metastatic GC compared with normal samples

**Figure 2:**
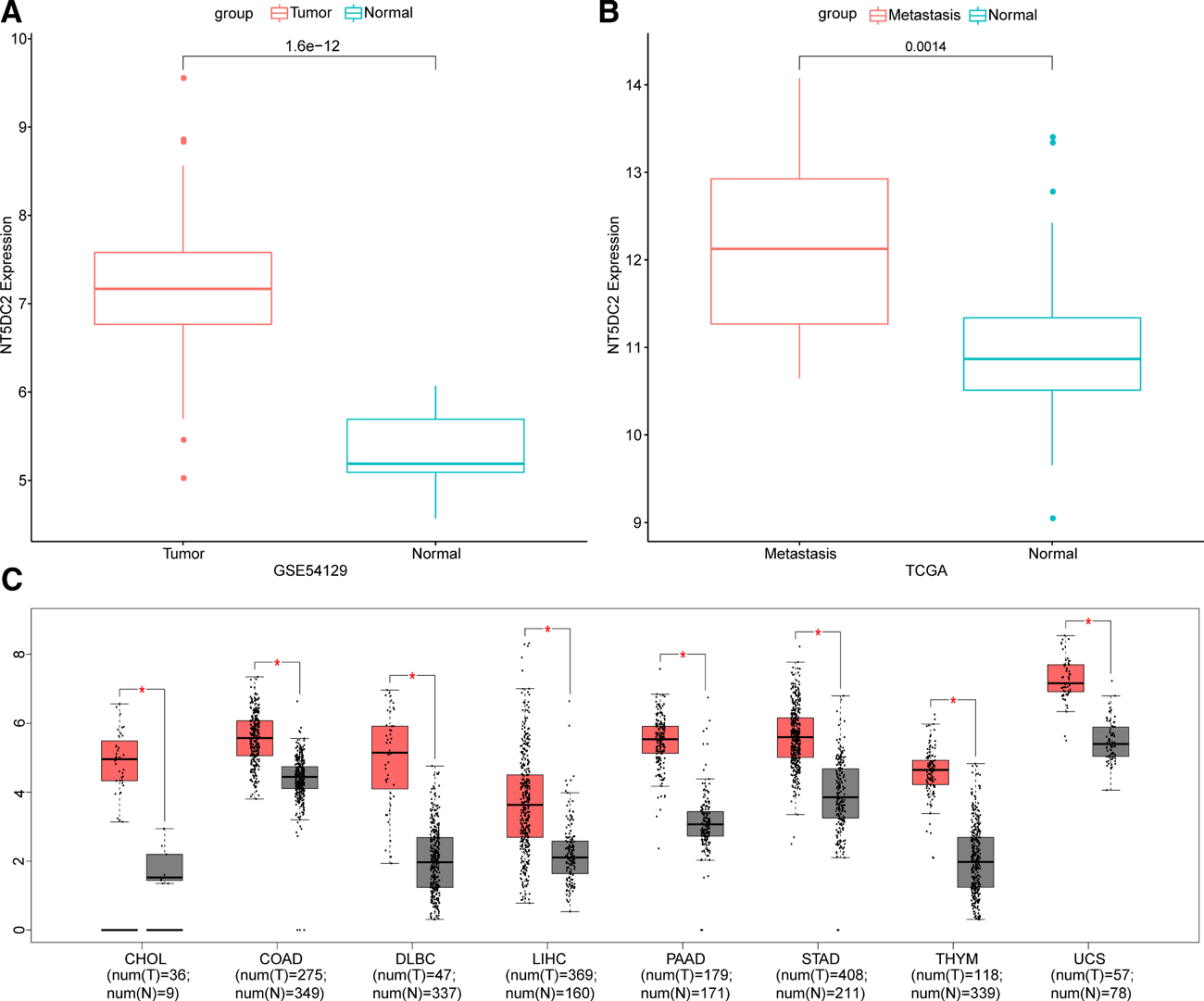
High NT5DC2 expression was observed in metastatic GC. (A,B) NT5DC2 expression in metastatic and normal GC samples, tumor and normal GC samples. (C) NT5DC2 expression in different cancers. GC = gastric cancer.

In GSE54129 dataset, our results showed that high expression of NT5DC2 was observed in primary GC samples compared with normal samples (Fig. 2A). Aside from this, in the TCGA database, NT5DC2 expression was up-regulated in metastatic GC samples when compared with normal samples (Fig. 2B).

Then, based on GEPIA (https://gepia.cancer-pku.cn/) database, we also examined the NT5DC2 expression in other cancers. Compared with corresponding normal samples, higher NT5DC2 expression was observed in stomach adenocarcinoma (STAD), thymoma (THYM) et al (Fig. 2C), which were consistent with trend of NT5DC2 expression in metastatic GC. Collectively, compared with normal samples, NT5DC2 was highly expressed in metastatic GC patients.

### 3.3. Metastatic GC patients with high NT5DC2 expression had a poor prognosis

**Figure 3:**
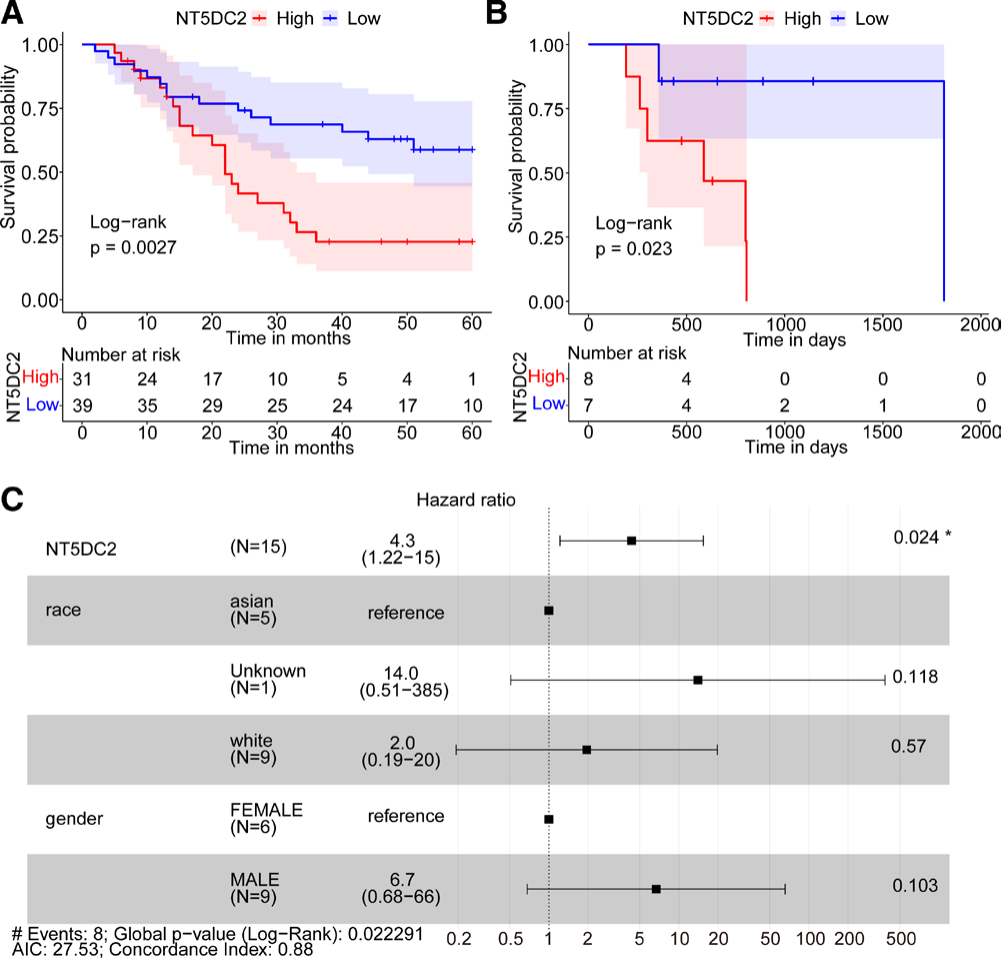
Metastatic GC with high NT5DC2 expression had a poor prognosis. (A,B) Kaplan–Meier survival curves of NT5DC2 expression in GSE57303, and metastatic GC cohort, respectively. (C) Forest plot from multivariate Cox regression analysis. GC = gastric cancer.

In GSE57303 dataset and TCGA database, we divided all GC samples into high and low groups according to the median NT5DC2 expression. KM survival analyses were performed to evaluate the prognostic value of NT5DC2 in GC. GC patients with high NT5DC2 expression in GSE57303 dataset and TCGA database had a poor prognosis (Fig. 3A,B).

Based on results of the KM plotter database (https://kmplot.com/). We found that high NT5DC2 expression resulting in GC patients of lymph node metastasis (OS N1 + 2 + 3HR = 1.58, *P* = .00067; PPS HR = 1.71, *P* = .00024), GC patients of negative HER2 (OS negative HER2 HR = 1.71, *P* = 3.20E-06; PPS HR = 1.67, *P* = .00045), GC patients of positive HER2 (OS positive HER2 HR = 1.69, *P* = 6.90E-05; PPS HR = 2.26, *P* = 6.80E-06) and GC patients of intestinal (OS intestinal HR = 2.28, *P* = 3.80E-07; PPS HR = 2.53, *P* = 1.30E-05) had poor OS and PPS (Table 1).

**Table 1:** The correlation between NT5DC2 and the survival of GC patients with distinct clinicopathological characteristics.

Clinicopathological characteristics	Overall survival(n = 881)			Progression-post survival(n = 503)		
	*N*	Hazard ratio (95% CI)	*P*	*N*	Hazard ratio (95% CI)	*P*
Gender						
Female	236	1.71 (1.2–2.44)	0.0026	149	1.75 (1.14–2.68)	0.0095
Male	544	2.15 (1.73–2.67)	2.40E-12	348	2.18 (2.11–3.66)	2.90E-14
Stage T						
2	235	1.57 (1.02–2.42)	0.038	196	1.92 (1.21–3.03)	0.0048
3	204	1.6 (1.13–2.27)	0.0075	150	2.09 (1.4–3.11)	0.00021
4	38	0.96 (0.41–2.24)	0.93	29	0.7 (0.28–1.74)	0.44
Stage N						
0	74	1.67 (0.65–4.31)	0.28	41	0.97 (0.31–3)	0.95
1 + 2 + 3	422	1.58 (1.21–2.05)	0.00067	337	1.71 (1.28–2.28)	0.00024
Stage M						
0	444	1.48 (1.12–1.96)	0.0059	342	1.98 (1.45–2.69)	1.00E-05
1	56	1.37 (0.77–2.46)	0.28	36	1.29 (0.63–2.63)	0.49
HER2 status						
Negative	532	1.71 (1.36–2.14)	3.20E-06	334	1.67 (1.25–2.22)	0.00045
Positive	343	1.69 (1.3–2.2)	6.90E-05	164	2.26 (1.57–3.25)	6.80E-06
Lauren classification						
Instestinal	320	2.28 (1.65–3.17)	3.80E-07	192	2.53 (1.64–3.89)	1.30E-05
Diffuse	241	1.14 (0.81–1.61)	0.44	176	1.24 (0.85–1.82)	0.26

The multivariate Cox regression analysis, including race and gender, was performed. Results showed that NT5DC2 expression is metastatic GC patients’ independent prognostic factor (Fig. 3C).

### 3.4. Potential NT5DC2-related pathways affecting GC metastases and prognosis

**Figure 4:**
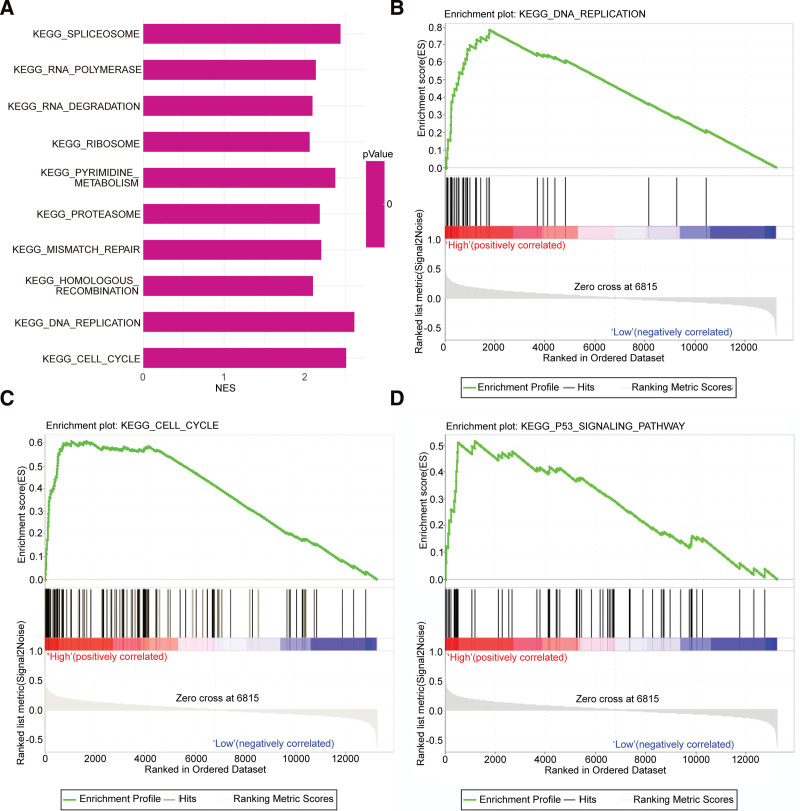
GSEA revealed NT5DC2-related pathways that might be associated with GC metastasis and prognosis. (A) The list of the top 10 most significantly activated pathways. (B) The DNA replication pathway. (C) The cell cycle pathway. (D) The p53 signaling pathway. ES = enrichment score, GSEA = gene set enrichment analysis, NES = normalized ES, NOM p-val = normalized *P* value.

In GSE14208 dataset, all metastatic GC samples were ranked from high to low based on the level of NT5DC2 expression. The top 20% of ranked samples were belonged to the high group, and the bottom 20% of ranked samples were belonged to the low group. Compared with the low group, there were 29 pathways significantly activated in the high group, as listed in Table S2 (Supplemental Digital Content, http://links.lww.com/MD/J815, which illustrates the 29 significantly activated pathways in high NT5DC2 expression samples compared with low NT5DC2 expression samples). The top 10 most significantly activated pathways are shown in Figure 4A. We displayed 3 significantly activated pathways (Fig. 4B,D), which might play a role in metastasis and development of cancer.

### 3.5. Immune cell infiltration and marker genes of immune cells in groups of high and low NT5DC2 expression metastatic GC samples

**Figure 5:**
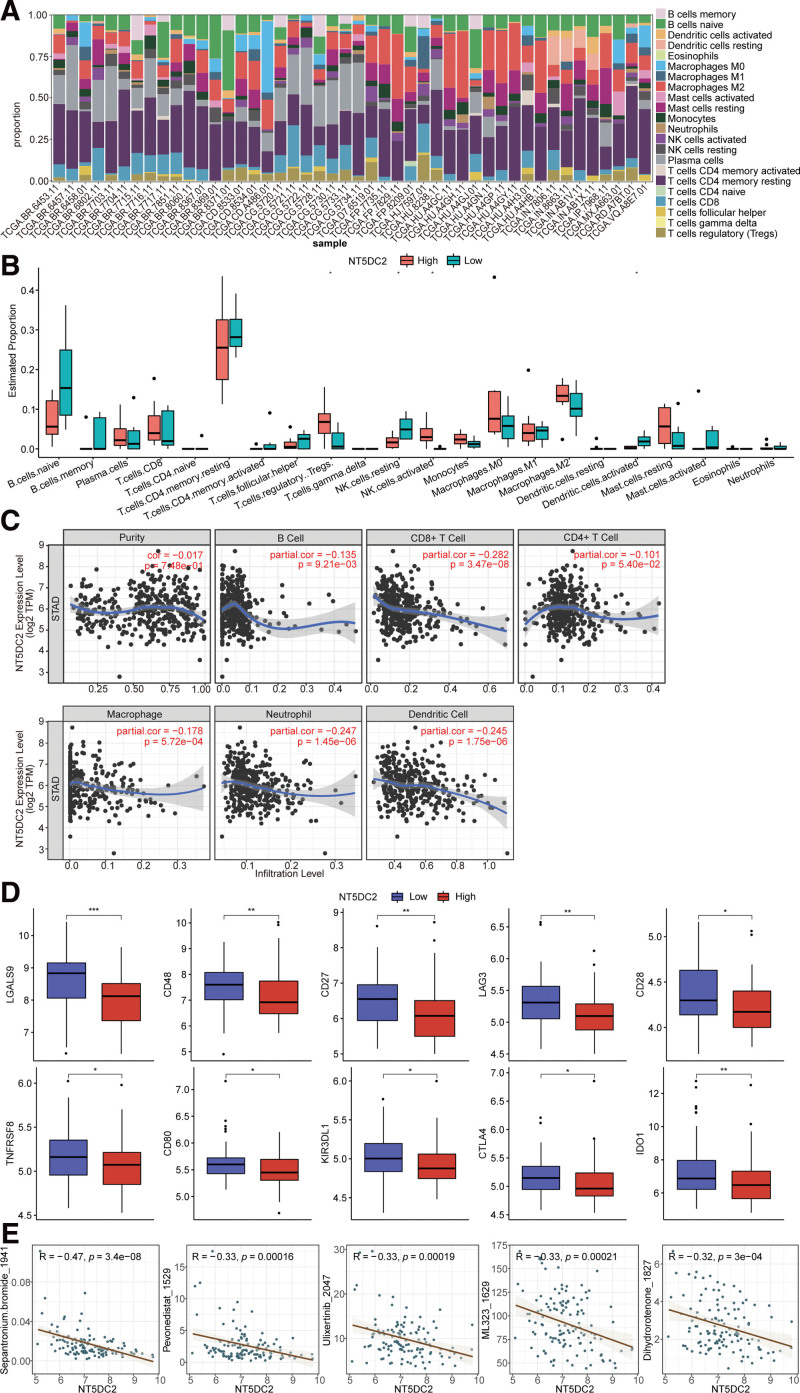
Results of immune cell infiltration. (A) The relative infiltration ratios of 22 kinds of immune cells. (B) Boxplot from different infiltration ratios of 22 kinds of immune cells in high NT5DC2 expression groups compared with low NT5DC2 expression groups. (C) Correlation of NT5DC2 expression with immune infiltration level in GC. (D) The expressions of LGALS9, CD48, CD27, LAG3, CD28, TNFRSF8, CD80, KIR3DL1, CTLA4, and IDO1 in high and low NT5DC2 expression groups in GSE14208 dataset. (E) The IC50 of Sepantronium.bromide_1941, Pevonedistat_1529, Ulixertinib_2047, ML323_1629, and Dihydrorotenone_1827 showed the strongest negative correlation with NT5DC2.

Considering the crucial impacts of the tumor immune microenvironment on GC metastasis, we analyzed of metastatic GC samples’ immune cell infiltration. The relative infiltration proportions of 22 kinds of immune cells were evaluated between metastatic GC samples and normal samples in TCGA database (Fig. 5A), and the results were listed in Table S3 (Supplemental Digital Content, http://links.lww.com/MD/J816, which illustrates the relative infiltration proportions of 22 types of immune cells in all samples). Thereafter, the relative infiltration ratios of 22 kinds of immune cells were evaluated between metastatic GC samples with high NT5DC2 expression and low NT5DC2 expression metastatic GC samples. All metastatic GC samples in TCGA database were divided into high and low expression groups based on the median NT5DC2 expression. The results showed that 4 types of infiltrating immune cells were significantly different between high and low NT5DC2 expression groups, including T cells regulatory, NK cells resting, NK cells activated, and dendritic cells activated (Fig. 5B).

Then, the correlation between NT5DC2 expression and immune cells’ critical marker genes was analyzed by TIMER database(http://cistrome.shinyapps.io/timer/). First, the correlations between NT5DC2 expression and infiltration of 6 types of immune cells were analyzed. Our results suggested significant negative correlations between NT5DC2 expression and 5 types of immune cells’ infiltration, including B cell (r=−0.135, *P* = 9.21e − 03), CD8 + T cell (r =−0.282, *P* = 3.47e − 08), macrophage (r=−0.178, *P* = 5.72e − 04), neutrophil (r=−0.247, *P* = 1.45e − 06), dendritic cell (r=−0.245, *P* = 1.75e − 06). As well, we found a negative correlation between NT5DC2 expression and CD4 + T cell(r =−0.101, *P* = 5.40e − 02)(Fig. 5C), but this correlation did not reach significance.

Thereafter, the correlations between the immune cells’ marker genes and NT5DC2 expression were analyzed. Significant negative correlations between NT5DC2 expression and 6 types of marker genes of immune cells were determined, including B cell (MS4A1, CD79A, and CD19), T cell (CD3D, CD3E, and CD2), CD8 + T cell (SCD8A), M2 macrophage (MS4A4A), natural killer cell (KIR2DL3, KIR3DL1, and KIR3DL2), monocyte (C3AR1 and CD86). Results also showed no significant correlations between NT5DC2 expression and the presence of regulatory T cells (Table 2). In GSE14208 dataset, we also analyzed the expression of 36 immune checkpoints, including CD276, CD244, CD200, CD86, CD80, CD48, CD40, CD28, CD27, TNFRSF25, TNFRSF18, TNFRSF14, TNFRSF9, TNFRSF8, TNFSF18, TNFSF15, TNFSF14, TNFSF9, TNFSF4, IDO2, IDO1, CD40LG, PDCD1 (PD1), CD274 (PDL1), PDCD1LG2 (PDL2), CTLA4, LAG3, TIGIT, BTLA, LGALS9, LAIR1, ICOS, TMIGD2, NPR1, KIR3DL1, and VTCN1 in high and low NT5DC2 expression groups, Compared with low NT5DC2 expression group, the expressions of LGALS9, CD48, CD27, LAG3, CD28, TNFRSF8, CD80, KIR3DL1, CTLA4, and IDO1 were significantly reduced in high NT5DC2 expression group (Fig. 5D). Finally, in GSE14208 dataset, we analyzed the correlation between NT5DC2 and IC50 of drugs, and found that NT5DC2 had prominent positive correlation with IC50 of 36 drugs and exhibited remarkable negative association with IC50 of 34 drugs, as listed in Table S4 (Supplemental Digital Content, http://links.lww.com/MD/J817, which illustrates the correlation between NT5DC2 and IC50 of drugs). The IC50 of Sepantronium.bromide_1941, Pevonedistat_1529, Ulixertinib_2047, ML323_1629, and Dihydrorotenone_1827 showed the strongest negative correlation with NT5DC2 (Fig. 5E). These results indicated that NT5DC2 might be a target gene for drugs in GC.

**Table 2:** In GC samples, the correlations between the immune cell marker genes and NT5DC2 expression.

Description	Gene markers	GC			
		None		Purity	
		Cor	*P*	Cor	*P*
CD8 + T cell	CD8A	–0.199	4.29E-05	–0.2	8.77E-05
	CD8B	–0.07	1.52E-01	–0.055	2.89E-01
B cell	MS4A1	–0.199	4.49E-05	–0.2	8.85E-05
	CD79A	–0.177	2.79E-04	–0.193	1.56E-04
	CD19	–0.124	1.17E-02	–0.124	1.58E-02
Natural killer cell	KIR2DL1	–0.07	1.56E-01	–0.054	2.93E-01
	KIR2DL3	–0.123	1.18E-02	–0.115	2.52E-02
	KIR2DL4	–0.087	7.54E-02	–0.066	2.00E-01
	KIR3DL1	–0.116	1.81E-02	–0.128	1.29E-02
	KIR3DL2	–0.124	1.14E-02	–0.116	2.36E-02
	KIR3DL3	–0.029	5.50E-01	–0.021	6.86E-01
	KIR2DS4	–0.079	1.06E-01	–0.093	6.91E-02
Monocyte	C3AR1	–0.114	1.98E-02	–0.127	1.36E-02
	CSF1R	–0.081	9.88E-02	–0.093	6.94E-02
	CD86	–0.104	3.47E-02	–0.111	3.11E-02
M2 Macrophage	CD163	–0.064	1.94E-01	–0.078	1.28E-01
	VSIG4	–0.061	2.16E-01	–0.064	2.13E-01
	MS4A4A	–0.153	1.75E-03	–0.159	1.94E-03
Regulatory T cell	CCR8	–0.071	1.49E-01	–0.073	1.55E-01
	FOXP3	–0.045	3.60E-01	–0.043	4.02E-01
T cell (general)	CD3D	–0.212	1.37E-05	–0.22	1.50E-05
	CD3E	–0.179	2.42E-04	–0.184	3.19E-04
	CD2	–0.19	9.77E-05	–0.194	1.44E-04

## 4. Discussion

Considering the significant effect that metastases can have on the prognosis of GC patients, we used bioinformatics techniques to discover a potential prognostic marker in GC from metastases-related genes and evaluated its potential impacts on metastatic GC. According to our findings, high levels of NT5DC2 expression in GC are closely correlated with metastases. Metastatic GC patients with high levels of NT5DC2 expression have a poorer prognosis.

NT5DC2 showed a high level of sequence similarity with NT5C2. Mutations of NT5C2 have been demonstrated to be associated with tumor cell metastasis, proliferation and chemotherapy resistance.^[17,18]^ Meanwhile, GC patients with metastases usually had a poor prognosis. A notable finding of our study was that high NT5DC2 expression is not only associated with metastases of GC but also with metastatic GC patients’ poor prognosis. In breast cancer, the NT5DC2 was upregulated and high NT5DC2 expression exhibited inferior prognosis of breast cancer patients, and the NT5DC2 expression was correlated with advanced Scarff-Bloom-Richardson grades and higher Nottingham Prognostic Index values.^[19]^ Schulze and colleagues studied the levels of NT5DC2 in 1925 nonsmall-cell lung cancer (NSCLC) patients (squamous cell tumor [48%], adenocarcinoma [35%], large cell histology [17%]), and discovered that the NT5DC2 mRNA levels were higher in squamous cell tumor compared to adenocarcinoma. And the high expression of NT5DC2 protein resulted in reduced the median overall survival of patients with stage I–III adenocarcinoma, while not in squamous cell carcinoma.^[20]^ An earlier study showed that NT5DC2 deletion represses the hepatic metastasis of NSCLC based on surface metastasis of liver and HE staining.^[13]^ In hepatic cellular cancer (HCC), high NT5DC2 expression HCC patients were also proved with a poor prognosis,^[21]^ which is also the case in glioma stem-like cells (GSCs).^[22]^ And the high expression of NT5DC2 was tightly correlated with higher tumor stage of HCC.^[23]^ Therefore, these findings suggest that high NT5DC2 expression may serve as an underlying metastatic GC patients’ prognostic biomarker.

Based on the results of GSEA, our study indicated that the cell cycle pathway is significantly activated in metastatic GC with high NT5DC2 expression. In the case of HCC and NSCLC, the knockdown of NT5DC2 has induced cell cycle arrest,^[13,21]^ indicating that the NT5DC2 has a correlation with cell cycle pathway. The previous studies also proved the role of cell cycle pathway in GC, where the cell cycle pathway is regulated by lncRNA CASC11, which promotes the migration and invasion of GC cells.^[24]^ Furthermore, it has been proven that the cell cycle pathway plays a key role in GC cell migration, and that NT5DC2 knockdown can also reduce NSCLC migration.^[13]^ Taken together, we suggested that NT5DC2 might induce the metastases process of GC by promoting the cell cycle pathway. However, the conclusive evidence needs further research in the future. Additionally, the p53 signaling pathway was also significantly activated in metastatic GC with high NT5DC2 expression. It has been reported that in NSCLC, the NT5DC2 expression had positive correlation with p53 protein and TP53 gene expression and its survival effect for lung adenocarcinoma was p53 dependent.^[20]^ However, p53 expression was up-regulated in NT5DC2 knockdown NSCLC cells.^[13]^ Likewise, in GC, the p53 signaling pathway was inhibited by low expression of neuronal pentraxin II (NPTX2). In addition, inhibition of the p53 signaling pathway suppressed the arrest of cell cycle progression,^[25]^ which triggered metastases of GC. Therefore, we obtain a bold hypothesis that high NT5DC2 expression induces GC to develop into metastatic GC, meanwhile p53 signaling pathway is activated during this process. However, Metastatic GC escaped the p53-related checkpoint due to high NT5DC2 expression. This will further prove that NT5DC2 can be a potential marker of GC patients’ prognosis. The assumption deserves our in-depth study to prove.

The recruitment of immune cells by tumor cells also promoted the cancer metastases.^[26]^ The results of TIMER database suggested that there are significant negative correlations between NT5DC2 expression and the infiltration of B cells, CD8 + T cell, macrophage, neutrophil, and dendritic cell. In breast cancer, NT5DC2 was positively related to CD4 + T cells infiltration, dendritic cells infiltration and was negatively linked to macrophages infiltration.^[19]^ In HCC, NT5DC2 expression exhibited positive association with infiltration of B cells, CD8 + T, CD4 + T cells, neutrophils, and dendritic cells.^[23]^ However, neutrophils released DNA meshes in response to infection, which, in turn, could promote GC cells metastases dependent on TGF-β signaling.^[27]^ In GC with USF1 deletion, *Helicobacter pylori* (Hp) infection strongly caused p53 loss.^[28]^ Combining our assumptions above, we speculated that pathogen infection inhibits the p53 signaling pathway, thereby promoting cell cycle. Finally, the cell cycle pathway coupled with the immune response of neutrophils promoted GC metastases. But this was caused by infection in the GC. In metastatic GC, we presumed GC metastases developed by high NT5DC2 expression induces cell cycle pathway. The correlation between high NT5DC2 expression and metastases in pathogen infection-induced GC has remained unknown. Furthermore, the negative correlation between NT5DC2 expression and neutrophil infiltration in metastases of GC should be further explored in the future. We also discovered that the expressions of immune checkpoints, including LGALS9, CD48, CD27, LAG3, CD28, TNFRSF8, CD80, KIR3DL1, CTLA4, and IDO1 were significantly reduced in high NT5DC2 expression metastatic GC samples group. Immune checkpoints are immune system negative regulators that play crucial roles in maintaining self-tolerance, avoiding autoimmunity, and protecting tissues from immune collateral harm.^[29]^ In cancer, immune checkpoints had a critical role in the tumor-related immune evasion and suppression.^[30]^ Immune checkpoint inhibitors (immunotherapy) stimulate the immune system via preventing cancer cells from activating immune suppressive pathways,^[31]^ which may improve the efficacy of tumor therapy. Moreover, the NT5DC2 exhibited remarkable negative association with IC50 of Sepantronium.bromide_1941, Pevonedistat_1529, Ulixertinib_2047, ML323_1629, and Dihydrorotenone_1827. Thus, we suspected that NT5DC2 might be a target gene for small molecule drugs and play significant role in response to immunotherapy in GC.

## 5. Conclusions

Taken together, the findings of this study represent the first report that demonstrates the potential role of NT5DC2 in GC patients. The prognosis of metastatic GC patients with high NT5DC2 expression was poor, and NT5DC2 may contribute to the metastases and prognoses of GC by mediating potential pathways, such as the p53 pathway and the cell cycle pathway.

## Author contributions

**Conceptualization:** Rula Sha, Jiaming Zhang.

**Data curation:** Rula Sha, Jiaming Zhang, Fanjie Meng, Getu Zhaori.

**Formal analysis:** Fanjie Meng, Getu Zhaori.

**Methodology:** Fanjie Meng, Getu Zhaori.

**Project administration:** Rula Sha, Jiaming Zhang.

**Software:** Fanjie Meng, Getu Zhaori.

**Visualization:** Fanjie Meng, Getu Zhaori.

**Writing – original draft:** Rula Sha, Jiaming Zhang.

**Writing – review & editing:** Rula Sha.

## Supplementary Material
